# A Diagnostic Challenge in Progressive Limb Weakness: A Case of Myasthenia Gravis With Atypical Distal and Cranial Nerve Involvement

**DOI:** 10.7759/cureus.93852

**Published:** 2025-10-05

**Authors:** Mohammad I Hafeez, Kasturi Krishnamoorthy, Nidhi Kakkar, Dhaval Odedara

**Affiliations:** 1 Department of Medicine for Older People, Stockport NHS Foundation Trust, Stockport, GBR

**Keywords:** atypical myasthenia gravis, autoimmune disorder, fourth nerve palsy, myasthenia gravis (mg), progressive lower limb weakness

## Abstract

Myasthenia gravis (MG) is an autoimmune neuromuscular disorder commonly characterised by fatigable muscle weakness, often presenting with ocular symptoms and proximal weakness, though atypical presentations can also occur, especially in patients with overlapping autoimmune conditions. Prompt diagnosis is essential to initiate effective treatment and prevent complications. We report a case of a 39-year-old male with type 1 diabetes mellitus (T1DM) who presented with a six-week history of progressive limb weakness, imbalance, and intermittent diplopia. Examination revealed bilateral distal upper limb weakness, a possible fourth left cranial nerve palsy, and generalised areflexia, without fatigable ptosis or bulbar involvement. MRI showed only minor cervical disc protrusions, and initial differential diagnoses included diabetic neuropathy, demyelinating disease, and mononeuritis multiplex. Neurophysiological testing confirmed a neuromuscular junction disorder, and acetylcholine receptor antibodies were significantly elevated, leading to a diagnosis of seropositive generalised MG. The patient was started on pyridostigmine and oral prednisolone, with clinical improvement. Further testing revealed autoimmune thyroiditis, and a CT of the thorax excluded thymoma. This report underscores the importance of recognising atypical presentations of MG, as distal limb weakness is not a usual manifestation and can mimic neuropathic disorders. Colinicians should consider MG as a differential diagnosis in patients with atypical neuropathy to ensure timely evaluation and management.

## Introduction

Myasthenia gravis (MG) is a chronic autoimmune disorder affecting neuromuscular transmission, most commonly mediated by antibodies against acetylcholine receptors (AChR) [1]. These antibodies reduce signal transmission across the neuromuscular junction, leading to characteristic fluctuating muscle weakness, typically affecting ocular, bulbar, and proximal limb muscles. The annual incidence of MG is approximately 8-10 per million population, with a bimodal age distribution: early-onset disease predominating in females under 40 and late-onset in males over 60 [1]. However, MG can occur at any age, and presentations vary widely. Classical features include ptosis, diplopia, dysarthria, and limb weakness that worsens with exertion and improves with rest [2]. However, atypical presentations - particularly those with predominant distal limb involvement or absence of fatigability - are diagnostically challenging and often mimic other neuromuscular or systemic diseases, such as Guillain-Barré syndrome or inflammatory myopathies.

MG is frequently associated with other autoimmune disorders, including autoimmune thyroid disease, type 1 diabetes mellitus (T1DM), and rheumatoid arthritis [3]. In such cases, the clinical picture may be further complicated by overlapping symptomatology and diagnostic confounders. For example, distal limb weakness in a diabetic patient may be attributed to diabetic polyneuropathy rather than an autoimmune neuromuscular disorder. This report discusses a diagnostically challenging case of MG in a middle-aged male patient with T1DM and newly diagnosed autoimmune thyroiditis, who presented with progressive distal weakness, imbalance, and subtle cranial nerve findings. The absence of hallmark MG features initially diverted clinical suspicion. However, a structured diagnostic approach, including neurophysiology and serology, ultimately confirmed the diagnosis and guided effective treatment.

## Case presentation

A 39-year-old left-handed male of Yemeni origin presented to the hospital with progressive motor symptoms. He had been living in the UK for three years and was unemployed at the time, previously training as a plumber. His medical history was significant for T1DM, diagnosed at age 21, with established diabetic retinopathy that had been treated with laser photocoagulation. The patient denied smoking, alcohol, or recreational drug use. Language barriers limited detailed history-taking, but with the help of an interpreter, a general timeline of symptoms was established. The patient had experienced joint pain in his distal interphalangeal (DIP) and proximal interphalangeal (PIP) joints, along with morning stiffness, beginning 2.5 years prior. About a year later, similar symptoms had developed in his feet, though no formal rheumatological diagnosis had been made.

Four to five months before presentation, he had developed binocular vertical diplopia, which had lasted about a month and resolved with the use of a prism. However, in late June 2025, his diplopia had recurred, and during a visit to Saudi Arabia, family members had noted right eyelid drooping. In early July 2025, he had begun experiencing weakness in the fourth and fifth fingers of his right hand, followed by similar symptoms in his left hand, making it difficult to perform grip and fine motor tasks. By mid-July 2025, he had developed a significant imbalance and difficulty walking, and during a casual football match, he had been unable to run and had trouble standing, resulting in falls. His condition had worsened by late July to early August 2025, leading to his hospitalisation with significant mobility limitations, generalised fatigue, and upper limb weakness.

**Figure 1:**
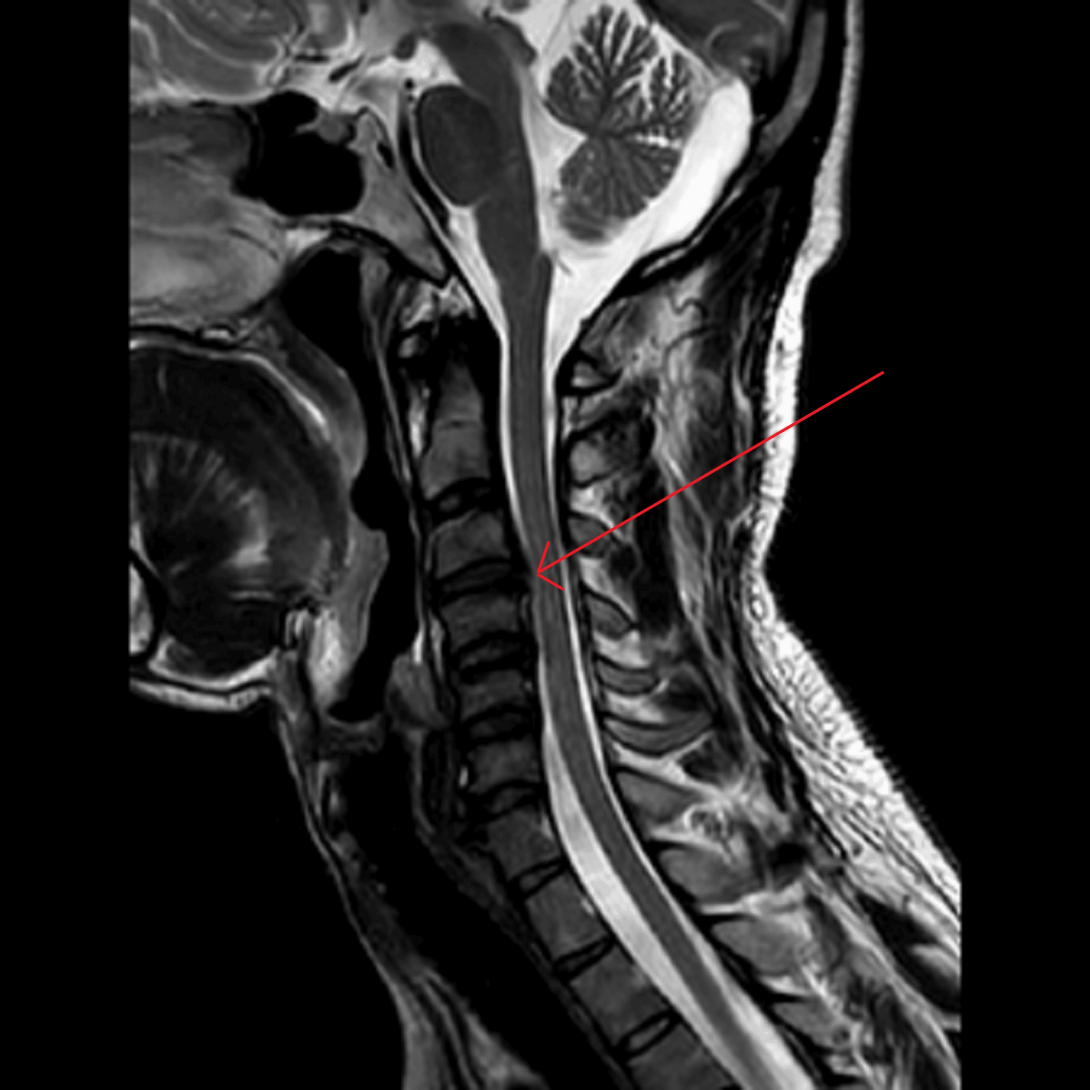
MRI brain and spine The arrow indicates a focal left para-central disc protrusion- small hernia, contacting with, and slightly compressing the spinal cord. The was no evidence of demyelination or cord signal change MRI: magnetic resonance imaging

On clinical examination, the patient had a subtle left fourth cranial nerve palsy and normal pupils and discs, with no fatigable ptosis. Although there was no objective facial weakness, the left side of his face appeared less expressive during conversation. There was no bulbar involvement. Motor examination revealed weakness in the distal upper limbs, particularly in finger extension bilaterally, and lower limb strength was 4/5 at the hip flexors, with slightly greater weakness on the right side. Reflexes were suppressed across multiple muscle groups, with only the biceps and knee reflexes present, while others were absent, and plantar responses were muted. Coordination testing was normal, and sensory examination was intact to all modalities. Gait testing showed mild imbalance on tandem walking, though the patient could walk on his toes and heels, and Romberg’s test was negative. There were no signs of cerebellar dysfunction or myelopathy. MRI brain and spine depicted a mild left-sided disc protrusion at C3-4 without any evidence of demyelination, as shown in Figure 1.

Investigations

Subsequently, further scans, lumbar puncture, and blood investigations were done. As shown in Table 1, the cerebrospinal fluid (CSF) was clear with normal cell counts, sterile cultures, and marginally elevated protein, with no malignant or infectious cells detected.

**Table 1:** Cerebrospinal fluid (CSF) analysis

Parameter	Result	Normal Range
Appearance	Clear, colorless	Clear, colorless
Protein	0.59 g/L	< 0.45 g/L
Glucose	6.7 mmol/L	Serum glucose 10.2 mmol/L
White cell count	<1 x 10⁶/L	<5 x 10⁶/L
Red cell count	<1 x 10⁶/L	<5 x 10⁶/L
Gram stain and cultures	Negative	Negative
Infection or malignant cells	None detected	None detected

Autoimmune screen was negative, except for markedly elevated anti-TPO antibodies with associated hypothyroidism (thyroid-stimulating hormone (TSH): 12 mU/L, low-normal free T4). Importantly, AChR antibodies were positive at 1.7 nmol/L, confirming the diagnosis of myasthenia gravis, as seen in Table 2.

**Table 2:** Serology ANA: antinuclear antibody; ANCA: antineutrophil cytoplasmic antibodies; APL: antiphospholipid antibodies; RhF: rheumatoid factor; C3/C4: complement components C3 and C4; Anti-CCP: anti-cyclic citrullinated peptide; TSH: thyroid-stimulating hormone; T4: thyroxine; Anti-TPO: antithyroid peroxidase; AChR: acetylcholine receptor

Test	Result	Normal range
Autoimmune screen		
ANA	Negative	Negative
ANCA	Negative	Negative
APL	Negative	Negative
RhF	Negative	Negative
Complement C3/C4	Normal	Normal
Anti-CCP	Negative	Negative
Thyroid function		
TSH	12 mU/L	0.4–4.0 mU/L
Free T4	Low-normal	10–20 pmol/L
Anti-TPO	>1300 IU/mL	<35 IU/mL
AChR antibodies	Positive at 1.7 nmol/L	<0.2 nmol/L

Inflammatory markers (erythrocyte sedimentation rate (ESR), C-reactive protein (CRP), infectious screening, and renal parameters were all within normal limits, as depicted in Table 3.

**Table 3:** Laboratory results ESR: erythrocyte sedimentation rate; CRP: C-reactive protein; HIV: human immunodeficiency virus; ACR: albumin-to-creatinine ratio

Test	Result	Normal range
Inflammatory markers		
ESR	7 mm/hr	<20 mm/hr
CRP	<5 mg/L	<5 mg/L
Infectious screen		
HIV	Negative	Negative
Hepatitis B/C	Negative	Negative
Syphilis	Negative	Negative
Urinalysis and renal screen		
ACR	Normal	<30 mg/mmol
Calcium-to-creatinine ratio	Normal	<0.3 mmol/mmol

CT head revealed no acute abnormalities, and CT thorax showed no mediastinal mass. As for neurophysiology, repetitive nerve stimulation demonstrated a decremental response at 3 Hz, consistent with impaired neuromuscular transmission. The pattern was characteristic of generalised MG.

Diagnosis

The patient was diagnosed with generalised MG based on clinical progression, neurophysiological findings, and a positive AChR antibody test. The presentation was atypical due to the absence of fatigable ptosis or bulbar symptoms, the presence of distal rather than proximal weakness, and subtle cranial nerve findings. Additionally, the background of autoimmune thyroiditis and T1DM suggested a possible diagnosis of autoimmune polyendocrine syndrome. Differential diagnoses, including diabetic polyneuropathy, mononeuritis multiplex, and demyelinating disease, were ruled out through comprehensive clinical evaluation, imaging, and electrophysiological testing.

Management

Management involved both pharmacological and supportive therapies. Pyridostigmine was initiated at 30 mg three times daily, with gradual titration to 60 mg QDS as tolerated. Prednisolone therapy began at 10 mg daily, increasing to 20 mg daily after one week, and levothyroxine 50 mcg daily was started for autoimmune hypothyroidism [1]. Propantheline was available to manage any potential cholinergic side effects from pyridostigmine, though it was not required initially. Supportive management included insulin adjustment under the supervision of Diabetes Specialist Nurses due to corticosteroid therapy, and ophthalmology review, where myasthenic ophthalmoplegia was suspected.

Cervical disc changes were deemed non-contributory, and outpatient spinal follow-up was arranged. Imaging for thymoma via CT thorax showed no thymic enlargement. The patient showed clinical improvement within 10 days of initiating treatment. Distal upper limb strength improved, particularly in finger extension bilaterally, and lower limb strength at the hip flexors increased to near normal (5/5). Reflexes, previously suppressed across multiple muscle groups, became more easily elicitable, and plantar responses normalised. The patient also reported subjective improvement in fatigue and overall functional ability, including easier performance of daily activities such as buttoning clothes and walking longer distances. He was discharged on pyridostigmine, prednisolone, and levothyroxine, with follow-up arranged with neurology, endocrinology, and ophthalmology.

## Discussion

This case illustrates the diagnostic complexity of MG when classical signs, such as fatigable ptosis, are absent [4]. The patient's presentation with distal limb weakness and subtle cranial nerve involvement without fatigable ptosis is atypical for MG and could be misleading, particularly in individuals with predisposing conditions like T1DM. MG is often underdiagnosed in its early stages or when it mimics peripheral neuropathy. Studies suggest that up to 20% of MG cases may present with non-ocular, non-fatigable, or isolated limb weakness [5], highlighting the importance of early neurophysiological testing to establish the diagnosis [6].

Additionally, this report emphasises the role of autoimmune clustering, as autoimmune thyroiditis and T1DM are components of autoimmune polyendocrine syndrome type 3, which is increasingly recognised in conjunction with MG [7]. The elevated anti-thyroid peroxidase (anti-TPO) levels [8] and abnormal TSH prompted further investigations, reinforcing the need for comprehensive autoimmune screening. While the patient’s cervical disc pathology raised concerns about potential structural cord involvement, the absence of myelopathy or radicular findings helped avoid unnecessary surgical intervention.

## Conclusions

MG can present with atypical features that resemble peripheral or structural neurological conditions. In this case, distal limb weakness and a lack of hallmark features delayed the diagnosis. However, systematic clinical assessment, neurophysiology, and serology enabled timely diagnosis and effective treatment initiation. Clinicians should maintain a high index of suspicion for MG in patients with autoimmune backgrounds, particularly when symptoms are progressive and multifocal. Early diagnosis and multidisciplinary management can lead to significant improvement and prevent long-term disability.
